# Data on corneal proteome and differentially expressed corneal proteins in highly myopic chicks using a data independent quantification approach

**DOI:** 10.1016/j.dib.2019.104478

**Published:** 2019-09-04

**Authors:** Byung Soo Kang, Thomas Chuen Lam, Jimmy Ka-Wai Cheung, King Kit Li, Chea-Su Kee

**Affiliations:** Centre for Myopia Research, School of Optometry, The Hong Kong Polytechnic University, Hong Kong SAR, China

**Keywords:** Myopia, Cornea, Corneal proteome

## Abstract

Myopia is an abnormal refractive status, explained by an excessive ocular lengthening mostly in posterior segments. Although growing evidence of anterior segments, specifically altered corneal geometries with biomechanical properties in myopes have been reported, the mechanism behind is poorly understood. We hereby prepared experimentally induced highly myopic chicks to investigate the molecular basis of corneal remodeling by applying a novel proteomic approach integrated with information dependent acquisition (IDA) and data independent quantification (SWATH-MS) analysis. As a result, differentially expressed protein biomarkers that might be involved in structural changes were screened based on the first of its kind unique chicken corneal proteome. All generated raw data from IDA and SWATH-MS are accessible at Peptide Atlas public repository (http://www.peptideatlas.org/PASS/PASS01410) for general release.

**Table:** Specifications Table

Subject area	Biology
More specific subject area	Corneal proteome in myopia development
Type of data	Table, Graph
How data was acquired	Quadrupole Time-of-Flight TripleTOF® 6600 mass spectrometer; SWATH Mass Spectrometry (SCIEX); searched against the UniProt database (Gallus gallus, organism ID: 9031)
Data format	Raw and analyzed
Experimental factors	High myopia induced by form deprivation for 7 days
Experimental features	Corneal proteins were extracted from highly myopic chicks. Corneal proteome libraries were generated and differentially expressed proteins were identified using information dependent acquisition (IDA) and data independent quantification approach (SWATH-MS), respectively.
Data source location	Centre for Myopia Research, School of Optometry, The Hong Kong Polytechnic University, Hong Kong SAR, China
Data accessibility	All raw data generated from IDA and SWATH-MS are accessible at Peptide Atlas public repository (http://www.peptideatlas.org/PASS/PASS01410) for general release. *Manuscript in prep*

**Table:** 

**Value of the data** • These data included first and the largest chicken corneal proteome generated from IDA analysis and offline high pH-reverse phased peptide fractionation technique, which might be specifically useful for future researches on corneal development using a chicken model. • The data of differentially expressed corneal proteins in the highly myopic chicken are potentially important for revealing the underlying mechanism of corneal structural changes during myopia development.

## 1 Data

**Fig. 1:**
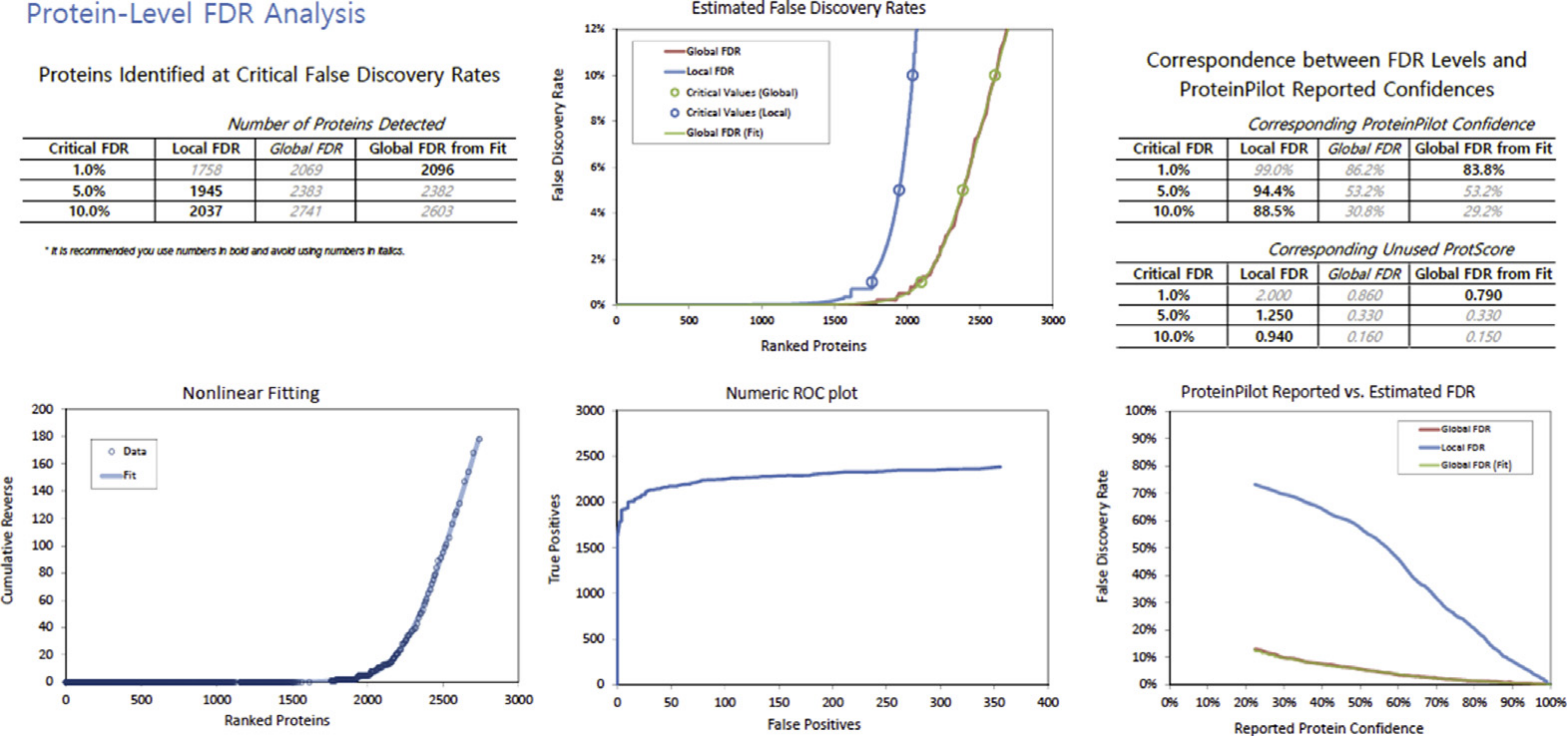
FDR analysis report of comprehensive IDA library from chicken corneas at protein level generated by ProteinPilot software.

**Fig. 2:**
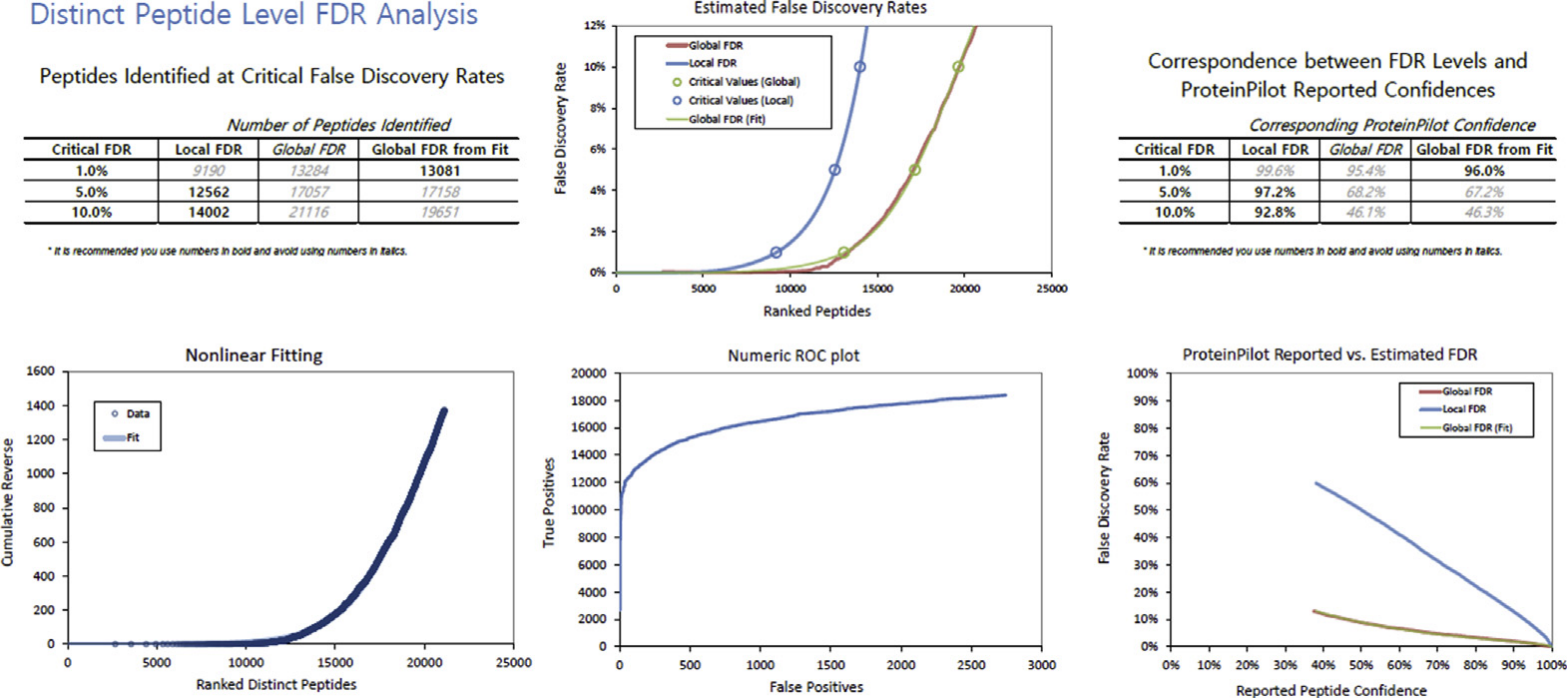
FDR analysis report of comprehensive IDA library from chicken corneas at peptide level generated by ProteinPilot software.

To test the effect of offline peptide fractionation and to increase the corneal proteome pool for SWATH data extraction, three ion spectral libraries (comprehensive, high-pH reverse phased fractionated, and unfractionated control) were produced for comparison. At 1% global false discovery rate (FDR) generated by ProteinPilot software (Version 5.0.1, Sciex Framingham, MA) with Paragon Algorithm [1], a total of 2096 non-redundant proteins (13081 peptides) were identified in comprehensive library (Table S1) with FDR analysis at protein (Fig. 1) and peptide levels (Fig. 2) shown, while 2016 and 1487 proteins were identified in fractionated (Table S2) and unfractionated control (Table S3) libraries, respectively. Differentially expressed proteins were screened by using SWATH-MS analysis with PeakView and MarkerView software (Sciex Framingham, MA). The list of proteins that passed cutting-off criteria of 2 minimum peptide matching, >1.2-fold differences with statistical significance of p < 0.05, and co-expressed in both comprehensive and fractionated libraries were highlighted in Table S4.

## 2 Experimental design, materials and methods

### 2.1 Animals

Eight White Leghorn chicks (Gallus gallus domesticus) were raised in the Centralized Animal Facility of The Hong Kong Polytechnic University. During the husbandry, chicks were given food and water ad libitum. The luminance of the animal room was maintained at 150 lux at chicks' eyes level with 12 hr:12 hr light-dark cycle. All experiments were conducted in accordance with the ARVO Statement for the Use of Animals in Ophthalmic and Vision Research and approved by the university's Animal Subjects Ethics Sub-Committee (ASESC16-17/22).

### 2.2 Treatments

Form deprivation (FD) myopia paradigm was induced on day 5 post-hatching (P5) by gluing detachable plastic-molded translucent diffusers on a Velcro over the feathers of right orbit. Left eyes were untreated and served as contralateral controls. After the 7-day treatment period, biometric measurements were performed as previously described in our work [2].

### 2.3 Tissue collections

FD treatment typically induces high myopia with higher intersubject variability. To minimize the potential effects due to intersubject variability on proteomics analysis, only chicks having high myopia (>20 D) with similar interocular changes of the corneal radius of curvature (<–7%) and axial length (>+9%) were selected (n = 3). After chicks were sacrificed by carbon dioxide asphyxiation, both eyes were enucleated and placed in chilled-PBS. Eyes were hemisected along the ora serrata using a razor blade. Ciliary body and crystalline lens were discarded gently, and anterior segments were washed briefly in chilled-PBS to remove aqueous humor. Corneal tissues of 4-mm diameter were collected using sterilized Biopsy Punches (Integra™, Miltex, U.S.). Collected tissues were rinsed again with chilled-PBS and snap-frozen in liquid nitrogen.

### 2.4 Homogenization

Each corneal tissue was loaded to a homogenizer (Precellys Evolution, Bertin Instrument, France) with 100 μL of EB2 lysis buffer [30 mM tris-HCI (pH 8.5), 7 M urea, 2 M thiourea, 2% (v/v) CHAPS, 1% (v/v) ASB14 with a complete protease inhibitor cocktail (cOmplete, Roche Roche Molecular Systems, U.S.)]. Samples were homogenized for 30 seconds at 6800 RPM with 2 cycles. The concentration of lysed protein was then quantified using the 2-D Quant Kit (GE Healthcare, U.S.) following the manufacturer's instructions.

### 2.5 Sample preparation

Based on the protein concentration, samples were diluted with EB2 lysis buffer to make equal concentration and volume (25 μL; 2.5 μg/μL) for faster sample preparation procedures. The identical amount of proteins from each sample were extracted and pooled as a purpose of building IDA spectral library. For protein reduction, 0.1 M dithiothreitol (DTT) was added (final concentration = 10 mM DTT) and incubated for 45 minutes in the 37 °C chamber. Afterwards, 0.2 M iodoacetamide (IAA) was mixed (final concentration = 20 mM IAA) and incubated for 20 minutes in a dark room. Samples were then suspended by 100% (v/v) acetone (volume = volume of sample x 4) and stored in −25 °C freezer overnight. The suspended samples were precipitated by 20-min centrifugation at 4 °C. The supernatant was discarded and samples were re-suspended by 80% (v/v) acetone, followed by centrifugation. After removing acetone, the tubes containing pellets were completely dried at the room temperature for 5 h. Then, 8 M urea dissolved in 0.1 M TEAB was added to tubes. To estimate the amount of trypsin for protein digestion, 3 μL sample was collected for protein assay (2-D Quant Kit, GE Healthcare, U.S.). Samples were digested with trypsin (1 μg per 25 μg protein amount), followed by incubating in a temperature-controlled (37 °C) shaking chamber (ThermoMixer, Eppendorf, Germany) for 18 hours. Afterwards, contaminants in the sample including detergent, buffer salts, organic modifier (e.g., DTT and urea) which could influence the quality of MS data [3], [4] were removed through cleanup kits (Oasis® HLB Sorbent Cartridge, Waters, U.S.), and re-suspended by adding 0.1% (v/v) formic acid.

### 2.6 Offline high-pH reverse phased peptide fractionation

The pooled corneal peptide samples in total 13 μg were fractionated by using a kit (Pierce™ High pH Reversed-Phase Peptide Fractionation Kit, Thermo Fisher Scientific, U.S.) according to the manufacturer's instructions. Briefly, columns in the kit were centrifuged to remove the solution and pack the resin material, followed by adding 300μL of 100% (v/v) ACN and 0.1% (v/v) TFA with centrifugation for column conditioning. For a flow-through fraction, 0.1% TFA was added to each sample and they were loaded into columns for centrifugation. The resins in the columns were then washed by adding water with centrifugation. Retained fractions were eluted by adding step gradient elution solutions with either 12.5% or 50% (v/v) ACN dissolved in 0.1% (v/v) TFA. Collected fractions were dried and re-suspended by adding 0.1% (v/v) formic acid. After performing peptide assay using a kit (Pierce™ Quantitative Colorimetric Peptide Assay, Thermo Fisher Scientific, U.S.), samples (2 μg each) were loaded for MS analysis.

### 2.7 LC-MS/MS configuration

A hybrid TripleTOF® 6600 quadrupole Time-of-Flight mass analyzer (Sciex Framingham, MA) connected to a nano LC415 was applied for proteomic data acquisition. Digested samples (2 μg) were loaded to trap column (350 μm × 0.5 mm, C18) for 15 minutes with a flow rate of 2 μL·min−1 with loading buffer (2% (v/v) ACN with 0.1% (v/v) formic acid). Then, samples were separated on the analytical column (100 μm × 30 cm, C18) in the mixture with a flow rate of 350 μL·min−1 using the gradient: 0–0.5 min: 5 %B, 0.5–90 min: 10 %B, 90–120 min: 20 %B, 120–130 min: 28 %B, 130–135 min: 45 %B, 135–141 min: 80 %B, 141–155 min: 5% with solvent A (2% (v/v) ACN with 0.1% (v/v) formic acid) and B (98% (v/v) ACN with 0.1% (v/v) formic acid). Samples were conveyed to TripleTOF 6600 through 10 μm SilcaTip electrospray emitters (New Objective, U.S.). For data acquisitions, a high-resolution TOF-MS scan mode with a mass range of 350–1500 *m*/*z* was set while 100–1800 *m*/*z* mass range was set for MS/MS. Intensity (ions greater than 125 cps) was one of the selection criteria for parent ions. Collision-induced dissociation was triggered by rolling collision energy. The ion accumulation time was set to 250 ms (MS) and 80 ms (MS/MS). For data independent acquisition (SWATH-MS), the instrument was set for variable isolation window in a looped mode over the mass range of 100–1800 *m*/*z* scans of 100 variable windows with an accumulation time of 30 ms.

### 2.8 Protein identification by IDA

Three types of reference proteome libraries were generated using the same pool sample with two technical replicates (2 μg each): fractionated peptide lysates with two different gradients (12.5% and 50% v/v ACN; see details in *Offline high-pH reverse phased peptide fractionation*) and unfractionated control. For generating comprehensive library, acquired MS raw data (.wiff) from both the fractionated and unfractionated samples were loaded and digitally integrated in ProteinPilot software to form the combined spectral ion library (i.e. comprehensive = fractionated + unfractionated control; fractionated = 12.5% + 50% v/v ACN; and unfractionated control). Protein identification was performed based on the Uniprot database (Taxonomy_9031_Gallus gallus) with searching parameters of trypsin digestion, iodoacetamide cysteine alkylation, and thorough search. The protein detection threshold (Unused Protscore) was set to > 0.05, equivalent to a confidence level of 10%, and the FDR analysis was also performed. Only screened proteins at 1% global FDR were considered for protein counts and further bioinformatics analysis.

### 2.9 SWATH-MS

Six samples (3 myopic and 3 contralateral fellows; 2 μg each) were loaded to MS with two technical replicates. Generated raw data (.wiff) were processed with PeakView (Version 2.1, Sciex, Framingham, MA) to extract relevant transitions of each identified peptide/protein. Two reference proteome libraries, produced from IDA analysis (comprehensive and fractionated) were used for matching the corresponding peptide fragment peaks. Then, a minimum of 10 peptides with high signal/noise ratios in-between 30 and 130 min of the run was selected for retention time calibration. Following parameters were set before peak extraction: 10 peptides per protein, 6 transitions per peptide, and a 10-min extracted-ion chromatogram (XIC) with 75 ppm width. Peptide confidence and FDR thresholds were given at 95% and 1% respectively. Resulting data were exported to MarkerView (Version 1.3.1, Sciex, Framingham, MA) for normalization using MLR method [5], followed by statistical analysis (unpaired *t*-test).
